# Evaluation of Suppressed Mite Reproduction (SMR) Reveals Potential for Varroa Resistance in European Honey Bees (*Apis mellifera* L.)

**DOI:** 10.3390/insects11090595

**Published:** 2020-09-03

**Authors:** Fanny Mondet, Melanie Parejo, Marina D. Meixner, Cecilia Costa, Per Kryger, Sreten Andonov, Bertrand Servin, Benjamin Basso, Małgorzata Bieńkowska, Gianluigi Bigio, Eliza Căuia, Valentina Cebotari, Bjorn Dahle, Marica Maja Dražić, Fani Hatjina, Marin Kovačić, Justinas Kretavicius, Ana S. Lima, Beata Panasiuk, M. Alice Pinto, Aleksandar Uzunov, Jerzy Wilde, Ralph Büchler

**Affiliations:** 1 INRAE, UR 406 Abeilles et Environnement, 84914 Avignon, France; 2 UMT PrADE, 84914 Avignon, France; benjamin.basso@inrae.fr; 3 Agroscope, Swiss Bee Research Center, 3003 Bern, Switzerland; melanie.parejo@ehu.eus; 4 Laboratory of Genetics, University of the Basque Country, 48940 Leioa, Spain; 5 LLH Bee Institute, 35274 Kirchhain, Germany; marina.meixner@llh.hessen.de (M.D.M.); uzunov@fznh.ukim.edu.mk (A.U.); ralph.buechler@llh.hessen.de (R.B.); 6 CREA Research Centre for Agriculture and Environment, 40141 Bologna, Italy; cecilia.costa@crea.gov.it; 7 Department Agroecology, Aarhus University, 4200 Slagelse, Denmark; per.kryger@agro.au.dk; 8 Departement of Animal Biotechnology, FZNH, 1000 Skopje, North Macedonia; sreten_andonov@yahoo.com; 9 Department of Animal Breeding and Genetics, SLU, 99104 Uppsala, Sweden; 10 INRAE, GenPhySE, 31326 Castanet-Tolosan, France; bertrand.servin@inrae.fr; 11 ITSAP, 75012 Paris, France; 12 Research Institute of Horticulture, 96–100 Skierniewice, Poland; malgorzata.bienkowska@inhort.pl (M.B.); beata.panasiuk@inhort.pl (B.P.); 13 Aspromiele, Regional Association of Honey Producers, 15121 Alessandria, Italy; gianluigi.bigio@aspromiele.it; 14 Institute for Beekeeping Research and Development, 013975 Bucharest, Romania; eliza.cauia@icdapicultura.ro; 15 Institute of Zoology, Academy of Sciences of Moldova, 2028 Kishinev, Moldova; valentinaceb@yahoo.com; 16 Norwegian Beekeepers Association, 2040 Kløfta, Norway; bjorn@norbi.no; 17 Faculty of Environmental Sciences and Natural Resource Management, Norwegian University of Life Sciences, 1430 Ås, Norway; 18 Ministry of Agriculture, 10000 Zagreb, Croatia; marica.drazic@mps.hr; 19 Department of Apiculture, Institute of Animal Science—Hellenic Agricultural Organization ‘DEMETER’, 63200 Nea Moudania, Greece; fhatjina@instmelissocomias.gr; 20 Faculty of Agrobiotechnical Sciences Osijek, Josip Juraj Strossmayer University of Osijek, 31000 Osijek, Croatia; komarin@pfos.hr; 21 National Bee Breeding Association, Virsuliskiu g 33, 05105 Vilnius, Lithuania; jkretas@gmail.com; 22 Centro de Investigação de Montanha, Instituto Politécnico de Bragança, Campus de Santa Apolónia, 5300-253 Bragança, Portugal; aslima@fc.ul.pt (A.S.L.); apinto@ipb.pt (M.A.P.); 23 CESAM-Ciências, Centro de Estudos do Ambiente e do Mar, Faculdade de Ciências da Universidade de Lisboa, Campo Grande, 1749-016 Lisboa, Portugal; 24 Faculty of Agricultural Sciences and Food, Ss. Cyril and Methodius University, 1000 Skopje, North Macedonia; 25 Apiculture Division, Faculty of Animal Bioengineering, Warmia and Mazury University in Olsztyn, Sloneczna 48, 10-957 Olsztyn, Poland; jerzy.wilde@uwm.edu.pl

**Keywords:** varroa, honey bee, SMR (suppressed mite reproduction), breeding, selection, resistance

## Abstract

**Simple Summary:**

The mite *Varroa destructor* represents a great threat to honey bees and the beekeeping industry. The opportunity to select and breed honey bees that are naturally able to fight the mite stands a sustainable solution. This can be achieved by evaluation of the failure of mite reproduction (SMR, suppressed mite reproduction). We conducted a large European experiment to assess the SMR trait in different populations of honey bees spread over 13 different countries, and representing different honey bee populations. The first goal was to standardize and validate the SMR evaluation method, and then to compare the SMR trait between the different populations. Our results indicate that it is necessary to examine at least 35 brood cells infested by a single mite to reliably estimate the SMR score of any given colony. Several colonies from our dataset display high SMR scores, indicating that this trait is present within the European honey bee populations. No major differences could be identified between countries for a given population, or between populations in different countries. This study shows the potential to increase selection efforts to breed *V. destructor* honey bee resistant populations.

**Abstract:**

In the fight against the *Varroa destructor* mite, selective breeding of honey bee (*Apis mellifera* L.) populations that are resistant to the parasitic mite stands as a sustainable solution. Selection initiatives indicate that using the suppressed mite reproduction (SMR) trait as a selection criterion is a suitable tool to breed such resistant bee populations. We conducted a large European experiment to evaluate the SMR trait in different populations of honey bees spread over 13 different countries, and representing different honey bee genotypes with their local mite parasites. The first goal was to standardize and validate the SMR evaluation method, and then to compare the SMR trait between the different populations. Simulation results indicate that it is necessary to examine at least 35 single-infested cells to reliably estimate the SMR score of any given colony. Several colonies from our dataset display high SMR scores indicating that this trait is present within the European honey bee populations. The trait is highly variable between colonies and some countries, but no major differences could be identified between countries for a given genotype, or between genotypes in different countries. This study shows the potential to increase selective breeding efforts of *V. destructor* resistant populations.

## 1. Introduction

The invasive parasitic mite *Varroa destructor* is one of the main drivers of honey bee (*Apis mellifera* L.) colony losses [1,2,3,4,5]. In Europe, where the mite was first introduced in the 1970s, varroosis is a major challenge for beekeeping [6,7,8]. In many countries, beekeepers frequently employ organic or synthetic acaricides to avoid losing their colonies. However, resistance to chemical treatments can evolve, rendering their application useless [9,10,11,12]. In addition, mite treatments with chemotherapeutics may cause adverse effects on the honey bees [13,14] and can leave residues in hive products [15,16].

To overcome these issues, a sustainable approach with a long-term perspective needs to be developed. Selecting and breeding honey bee stock able to counteract the varroa mite would contribute to such a strategy. Populations of honey bees capable of surviving varroa infestations without treatment are well-described, and detailed investigations have provided insights regarding the underlying mechanisms [17,18,19,20,21]. Investigations of relevant traits to be utilized for selection towards increased varroa resistance already started in the 1990s, and since then, several breeding programs have yielded promising results [22,23,24,25]. In this paper, we use the term resistance according to the definition of [26], since the fitness of the mite is compromised.

Among the numerous mechanisms known to limit varroa mite population growth, suppression of mite reproduction (SMR) seems to play an important role and has been observed in the naturally resistant populations from Gotland and Avignon [27,28]. This trait first described by [29] refers to mites that enter a brood cell to complete their reproductive cycle, but eventually do not produce any mature and mated female progeny. Suppression of mite reproduction is considered a colony-level trait and defined by the proportion of worker brood cells containing non-reproducing mother mites. The trait was found to be heritable [30] and was utilized in U.S. breeding programs since the late 1990s [31,32,33]. Such lines are used by several commercial beekeepers, but no large-scale beekeeping practices that abstain from regular varroa treatments have been reported so far. In European selection programs, however, the trait has not yet received much attention, and data about the variability of mite reproductive success and the distribution of the trait in nonresistant, managed honey bee populations across Europe are missing. Moreover, when initiating any breeding attempts on the SMR trait in a given environment, it is important to screen the local population for the presence and variability of SMR and thus evaluate its potential for selection.

Several different mechanisms may trigger the SMR phenotype and may originate from host and/or parasite features. SMR can indirectly result from adult bee behaviors such as varroa-sensitive hygiene (VSH) or recapping behaviors [34,35]. Mechanisms of physiology or behavior of the brood may also influence the ability of varroa to reproduce [36,37,38], but remain unknown. Parasite features may also influence varroa reproduction, such as variation in mite genotypes [39,40], or the physiological status of mites invading cells.

To provide baseline data for regional breeding programs, we initiated a common study to evaluate the present of SMR in local European honey bee populations according to geographical locations and genotypes. We developed a common protocol to accurately identify the proportion of non-normally reproducing mites in a given colony, and to ensure data compatibility among the participants. We conducted simulations to estimate the accuracy of the SMR estimates and optimize future research. We also discuss potential mechanisms that may be present in different breeding stocks, such as behavior of the bees, physiological features of the brood, or parasite features.

## 2. Materials and Methods

### 2.1. Honey Bee Colonies and Sampling Strategy

This study was conducted by 17 laboratories in 13 European countries (Table 1) during the summer and fall of 2015 and 2016. A total of 414 colonies, distributed in 68 apiaries and managed by the participating institutes or by partner beekeepers, were evaluated.

The experimental colonies originated from stock maintained at the participating institutes and, according to the expert opinion of the respective experimenters, belonged to European subspecies, local hybrids, or local populations of *Apis mellifera* (*A. m. carnica*, *A. m. caucasica*, *A. m. cecropia*, *A. m. iberiensis*, *A. m. ligustica*, *A. m. macedonica*, *A. m. mellifera*, *A. m. carpatica*, Buckfast, and hybrids of Carnica, Ligustica, and Mellifera), referred hereafter as “genotypes”. While no genetic screening was employed to confirm subspecies origin, the populations represent distinct local populations and can be considered as different genotypes. Sampled colonies were randomly chosen from each local population. We also included 23 colonies from two populations that were preselected for varroa resistance: *A. m. mellifera* hybrids from a French varroa-surviving population [17] and colonies containing *A. m. mellifera* hybrid queens artificially inseminated with semen collected from colonies of a VSH (Varroa sensitive hygiene) breeding program (Danka et al., USDA Baton Rouge, USA) [31]. A summary of participating institutes, laboratories, respective genotypes, and the number of investigated samples is presented in Table 1.

### 2.2. Evaluation of Mite Non-Reproduction

A reliable assessment of mite offspring stages requires a considerable level of knowledge, skill, and experience of the evaluator. In particular, female protonymphs and male offspring are difficult to differentiate [6,41], and their correct identification can be challenging. To improve and promote the reliability of measurements in the present study, a standardized protocol was developed, including detailed photographs of the respective developmental stages of bee pupae and mite offspring, which was shared among all participants of the experiment [42]. In addition, all participants of the study had the opportunity to gain experience by attending a training workshop where the scoring method was demonstrated and practiced.

To determine the proportion of mites that had infested brood cells but failed to reproduce, a frame containing capped, worker brood at late developing stages (pupae with purple eyes and white body or older, i.e., at least 7 days postcapping) was sampled from each colony and assessed in the laboratory. Frames were dissected fresh when possible, or after storage (for 1–6 months) at −20 °C.

**Figure 1:**
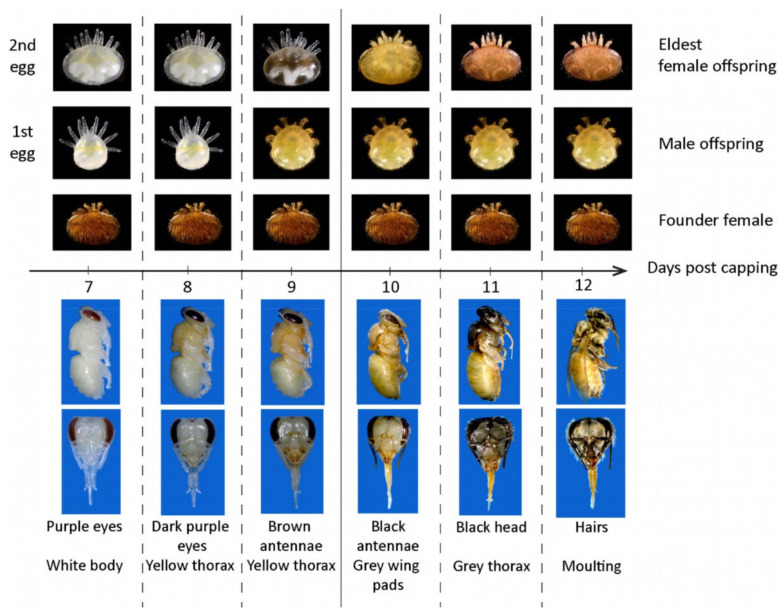
Staging chart used to determine the reproductive success of female varroa mites. Photographs show the average appearance of the development of mite progeny (first two eggs—upper part) in relation to the bee pupal stage (lower part). For bees, the main characteristics used to determine each stage are indicated below the photographs. For mites, the normally expected stage of the eldest female and male offspring are indicated above the photographs. If the eldest progeny was at a younger stage than the one corresponding to the illustration of a given bee stage, then the foundress mite was classified as non-reproducing. The solid line placed between day 9 (bees with colored thorax, and white wing pads) and 10 (bees with grey wing pads) days postcapping separates the period before and after which we should expect adult female varroa offspring. Source: Beebreeding.net.

On each frame containing combs, brood cells were randomly selected and carefully opened under a stereo-microscope. If a cell was infested, the developmental stage of the pupa was scored. Three stages were distinguished, according to morphological characteristics: <7 days postcapping (pupae with eyes lighter than dark purple), 7–9 days postcapping (pupae with dark eyes and light body coloration), and 10–12 days postcapping (pupae with dark body coloration). The main criterium to differentiate between postcapping day 9 and 10 was the presence of grey wing pads at day 10. In addition, the composition of the mite family was carefully assessed by recording the number of foundress mites, the stage of the eldest female offspring, and, optionally, the presence and stage of male offspring. A table describing the normal development of mite offspring with regard to the development of bee pupae was used to determine whether the foundress mite would have produced at least one mated daughter by the time the bee emerged from the cell (Figure 1).

Only single-infested brood cells (one foundress mite only) containing pupae older than 7 days postcapping were scored, as it is impossible to determine the success of mite reproduction in earlier stages and in case of multiple infestations. A foundress mite was considered reproductive if it was accompanied by offspring at least as old as described in the chart (Figure 1). In the 7–9 days postcapping stage, normally reproducing mites have at least one deutonymph or adult son and one deutonymph daughter. In the 10–12 days postcapping stage, normally reproducing mites have at least one adult son and one adult daughter. The foundress was considered non-reproductive if the offspring was younger (delayed reproduction), if the male was missing (no male), or if no offspring was present (infertile mite). Further details on the protocol and more illustrations can be found at www.beebreeding.net.

On each comb, cells were dissected until at least 10, or if possible 35, single-infested cells were identified. Brood infestation rate was estimated as the number of cells containing mites over the total number of screened cells, and the SMR score was calculated as the proportion of infested cells containing non-reproducing mites.

### 2.3. Simulation Analyses

Theoretical calculations were performed to determine the influence of the number of infested cells opened on the precision of SMR estimation. Given a number of single-infested cells (SIC) opened (*i*) and the true SMR value (*s*), the observed number of non-reproducing cells (*r*) can be considered as random. To account for the sampling variability in *r*, the sampling process was averaged over all possible values of *r*:

(1) $$P\left(\tilde{s}|i,s\right)=\overset{i}{\underset{r=0}{\sum }}P\left(\tilde{s}|r,i\right)P(r|i,s)$$

where $P\left(\tilde{s}|r,i\right)$ follows a beta distribution with parameters (1+*r*, 1+*i*-*r*) and P(r|i,s) a binomial distribution of parameters *i* and *s*. Hence, the distribution of SMR estimates ($\tilde{s}$) is a mixture of beta distributions. These distributions were derived for varying values of SIC and SMR.

In a second step, to improve the estimation of SMR, all colonies were modelled jointly to gather strength across colonies and get more robust estimates of SMR for colonies that have been evaluated with few SIC. This hierarchical approach assumes that true SMR values of colonies arise from a common beta-distribution *Beta (a, b)*. In a Bayesian setting, the beta distribution is the prior distribution on SMR. Because many colonies were evaluated on SMR, the parameters of the prior distribution can actually be learned from the data using an empirical Bayes strategy: first, raw values of the SMR estimates are obtained (*r*/SIC), then, based on the global distributions of these estimates, prior distribution parameters are estimated via maximum likelihood. Finally, posterior means are calculated for all colonies and used as robust estimates of SMR.

### 2.4. Statistical Analyses

All statistical analyses and figures were generated in the R environment (Version 3.3.1). Colonies were considered as individuals. The relationship between the brood infestation rate and the SMR score was tested using Pearson correlation tests. Due to the nature of the experimental design and the data (proportion data), analyses were performed using generalized linear models (GLM—package *lme4*), and as a quasi-distribution was fitted, Fisher tests were subsequently used. A generalized linear mixed-effects model (GLMM) was used in the case of the comparison within the *carnica* group. To account for the fact that colonies could belong to the same apiary in this latter case, the identity of apiaries was included as a random factor, along with treatment effect as a fixed explanatory variable (genotype, country, or SMR level). Pairwise comparisons of factor levels were performed using post-hoc tests (fdr or Tukey).

## 3. Results

### 3.1. Variability of SMR in Different European Countries

**Figure 2:**
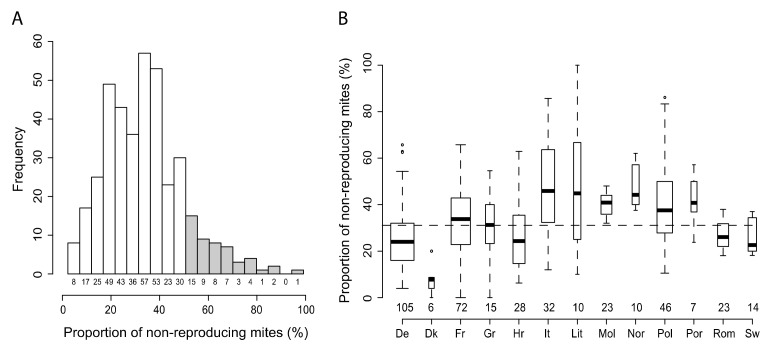
Distribution of the suppression of mite reproduction (SMR) score within Europe. (**A**) Histogram of distribution of the total data and (**B**) SMR scores by country for each of the 13 sampled countries. Grey histogram bars indicate colonies with a varroa-resistance potential (SMR score ≥50%). Boxplot widths are proportional to the sample size which is indicated below each bar or plot. The dashed line represents the average SMR score among the sampled European countries. ° Indicate data points distributed outside 1.5 interquartile space.

Descriptive analysis based on the complete dataset (≥10 SIC per colony, *n* = 414 colonies) resulted in an overall average SMR score of 32.8% ± 16.8 and a median of 31.4%. The SMR score of colonies that had not been preselected for varroa resistance varied between 0 and 100%. Overall, most colonies displayed a score between 0 and 50%, with 15.9% of the colonies showing an SMR score equal to or greater than 50% (Figure 2A).

However, the median and range of SMR scores varied substantially between the different countries where the study was performed, ranging between 4.0% ± 10.6 (Denmark) and 24.5% ± 16.9 (Italy) (Figure 2B).

### 3.2. Protocol Improvement to Estimate a Reliable SMR Score

**Figure 3:**
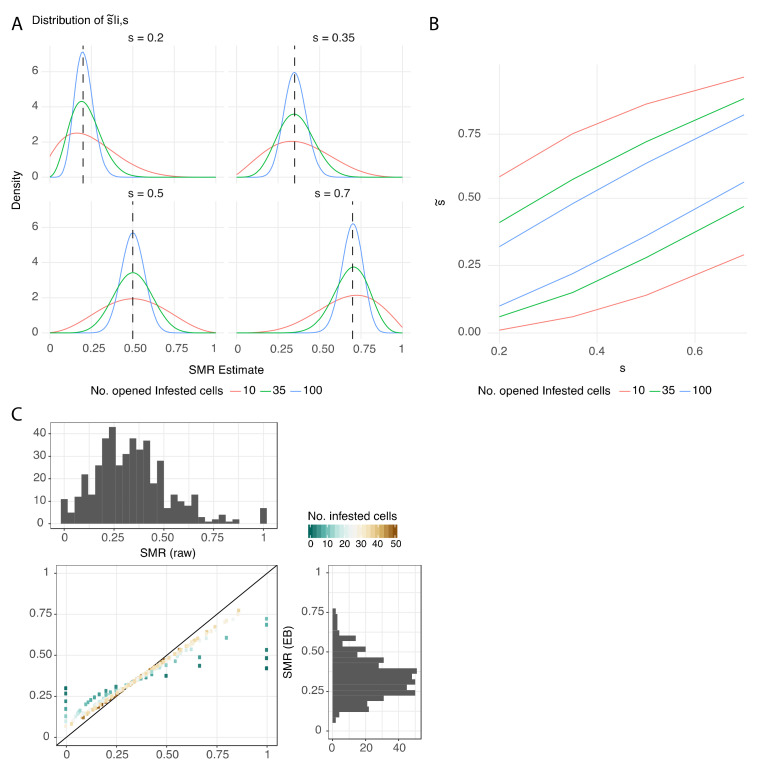
SMR estimates reliability. (**A**) Density of distribution of the SMR estimate, depending on the SMR value (s = 0.2, 0.35, 0.5 or 0.7) and the number of opened single-infested cells (SIC, no. of SIC = 10, 35, or 100). (**B**) Precision of the SMR estimate (95% quantiles), depending on the SMR estimate. Three examples of SIC numbers are represented (10, 35, and 100) to illustrate the fact that using 10 SIC does not give a reliable SMR estimate. (**C**) “Empirical Bayes” (EB) distribution with the initial distribution of raw SMR values (top) and comparison to the EB estimates. The distance to the identical distribution (black line) is acceptable when the number of SIC is greater than 30 (pale brown).

Estimation of the true SMR score varies depending on the true SMR score itself and on the number of cells opened (Figure 3A). The variation is nonsymmetrical for small and large values of SMR when SIC is small (<10). For instance, a high SMR score (0.7) is more likely under- than overestimated, while a low SMR score (0.2) is more likely over- than underestimated. Irrespective of the SMR score there is high variability in its estimate in particular for small SIC. For instance, with 10 SIC and a true SMR value of 0.35, the measured SMR score can be overestimated to be higher than 0.5 with a probability of 24%, and it can even be overestimated to be higher than 0.7 with a probability of 4%. The variability was further quantified by looking at the cumulative density function of the SMR estimate (Figure 3B). It is clear that 10 SIC is not sufficient to obtain an SMR score with a satisfying variability (max. ± 30% of raw variability, Table S1). Thirty-five SIC stands as a minimum requirement, with more acceptable variability (max. ± 20% of raw variability). This result is confirmed by the Empirical Bayes distribution, where the distance to the identical distribution is acceptable when the number of SIC is greater than 30 (Figure 3C). In this shrinkage approach, it appears clearly that colonies assessed with only a few SIC (<15) have estimated values highly shrunk compared to their raw value, which highlights once again their low reliability. SMR analyses using 100 SIC would provide ideal reduced variability (ca. ± 12%), but practical aspects in the field and lab must be considered to evaluate the feasibility of such a standard.

### 3.3. Variability of SMR in Different Honey Bee Genotypes

**Figure 4:**
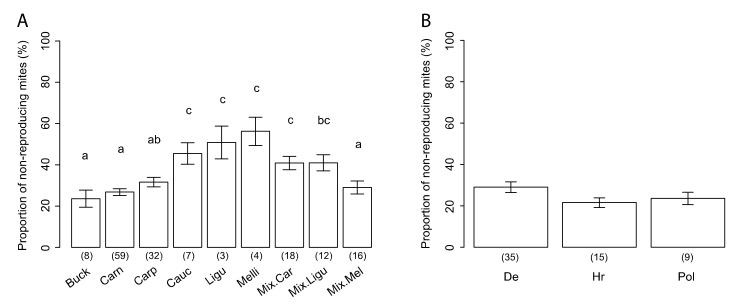
Variation of the SMR score according to the honey bee genotype. (**A**) Scores in the nine different genotypes. (**B**) Scores in the *A. m. carnica* genotype, sampled in three different countries. n-values are indicated below each bar. Different letters indicate significant differences between groups. Buck, Buckfast; Carn, Carnica; Carp, Carpatica; Cauc, Caucasica; Ligu, Ligustica; Melli, Mellifera; mix, hybrid; De, Germany; Hr, Croatia; Pol, Poland.

Based on the data of colonies with at least 35 SIC, significant differences of SMR were observed between the different genotypes (Figure 4A—GLM: F = 6.758, *p* < 1.49 × 10^−7^): *A. m. caucasica*, *A. m. ligustica*, *A. m. mellifera*, together with Carnica and Ligustica hybrids had higher SMR scores than *A. m. carnica*, Buckfast, *A. m. carpatica*, and Mellifera hybrids.

*A. m. carnica* genotypes were sampled in three different countries (Germany, Croatia, and Poland). Colonies from Germany tended to exhibit higher SMR scores than those from Croatia and Poland, but the differences were not significant (Figure 4B—GLMM: χ^2^ = 4.85, *p* = 0.088). It is important to note that within a given genotype and within a given country, the variability of the SMR score can be particularly high. For instance, in Germany, *A. m. carnica* bees displayed SMR scores between 14.2% and 65.7%.

**Figure 5:**
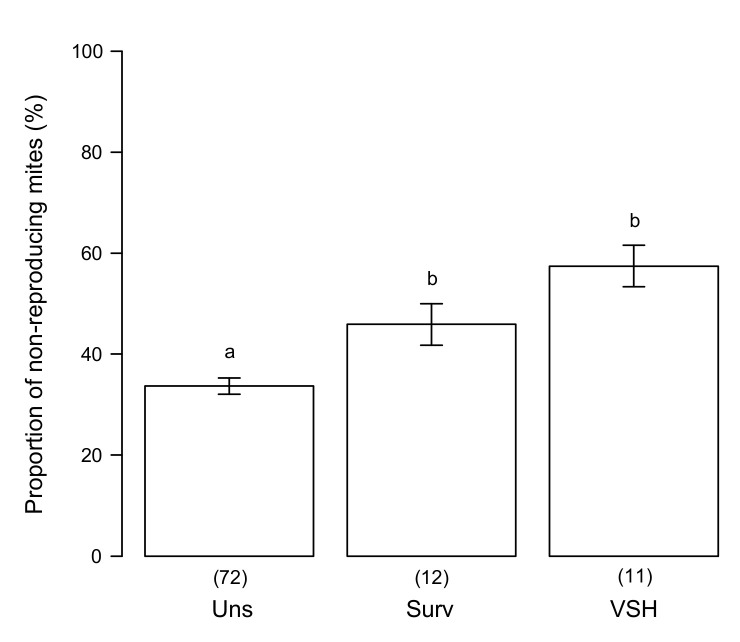
Effect of preselection for varroa resistance on the SMR score. Adjusted mean (± standard error) SMR scores between an unselected population (Uns, unselected) and two populations selected using varroa-resistance-related criteria (survival: Surv, VSH: VSH). Sample sizes are indicated below each bar and different letters indicate significant differences between groups.

Colonies from preselected populations with increased varroa resistance displayed a significantly higher SMR score than unselected ones (Figure 5—GLM: F = 86.32, *p* = 6.77 × 10^−7^). 

### 3.4. Putative Mechanisms for SMR

**Figure 6:**
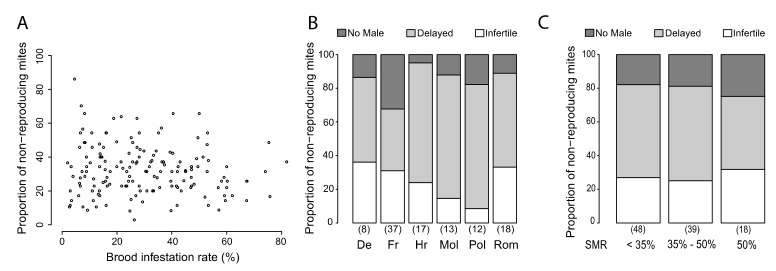
Putative mechanisms for SMR. (**A**) Relationship between brood infestation rates and SMR scores (*n* = 159). (**B**) Analyses of causes for non-reproduction (*n* = 105) according to the country, and (**C**) according to the level of the SMR score. N-values are indicated below each bar.

Based on the data of colonies with at least 35 SIC (*n* = 159 colonies, from 10 different countries), no significant correlation could be identified between the rate of brood infestation and the SMR score (Figure 6A—t = −1.48, *p* = 0.14).

In addition, the reason for classifying an infested brood cell as non-reproductive was investigated based on the following mite physiological criteria: delayed reproduction, missing male, or infertile (no offspring). These three criteria were analyzed in six countries (Germany, France, Poland, Croatia, Moldova, and Romania) where colonies containing at least 10 non-reproducing mites could be identified (Figure 6B). The proportion of cells being classified as non-reproductive due to the absence of a male was significantly different between countries (GLM: F = 6.68, *p* = 3.33 × 10^−8^). In comparison to Germany, a higher proportion of cells with no male varroa was identified in France (Fisher post-hoc: t = −7.38, *p* = 5.2 × 10^−11^). Similarly, the proportion of cells being classified as non-reproductive due to infertility was significantly different (GLM: F = 6.56, *p* = 2.59 × 10^−5^). In comparison to Germany, Moldova (Fisher post-hoc: t = −3.25, *p* = 0.0016) and Poland (Fisher post-hoc: t = −3.98, *p* = 0.00013) had a lower proportion of infertile varroa infested cells.

The cause for reproduction failure of the foundress varroa females was further investigated on the same dataset in relation to the degree of SMR of the colony, which was categorized as low (<34%, less than the average SMR in the study), medium (35–49%), or high (>50%, corresponding to potentially resistant colonies) (Figure 6C). Similar proportions of non-reproducing cells due to the absence of a male were detected in all three SMR categories (GLM: F = 1.11, *p* = 0.33). A similar result was found for the proportion of infertile cells (GLM: F = 0.75, *p* = 0.48) and for the proportion of delayed cells (GLM: F = 2.25, *p* = 0.11). Overall, no correlation was found between the SMR score and the proportion of absent males (Pearson: t = 1.52, *p* = 0.13), infertile foundresses (Pearson: t = 0.62, *p* = 0.54), or delayed reproduction (Pearson: t = −1.65, *p* = 0.10).

## 4. Discussion

### 4.1. SMR Trait in European Colonies

Suppression of mite reproduction (SMR) has been recognized as an important trait for survival in naturally resistant honey bee populations [24,28] and has been successfully implemented in breeding programs in the U.S. [22,31,43,44]. In contrast, beyond investigations in naturally resistant populations [21,27,28], the distribution of the SMR trait in European honey bee populations has not yet received major scientific attention. Most breeding efforts for varroa resistance in Europe have, until recently, relied on the introduction of nonlocal resistant stock, however, these attempts have not been successful [28,45]. A contributing factor to the failure of such attempts could result from genotype-environment interactions [46] favoring colonies’ adaptation to the prevailing environmental conditions [47]. When initiating any breeding attempts on the SMR trait in a given environment, it is important to screen the local population for the presence and variability of SMR and thereby evaluate its potential for selection.

In the present study, we screened 414 colonies across the entire European continent to provide a comprehensive dataset that describes the underlying variation of mite non-reproduction in Europe, which may serve as baseline data for selection decisions in prospective breeding programs. To obtain a first general overview on the distribution and variability of SMR in Europe with manageable input of labor, each participating laboratory investigated its colonies based on at least 10 single-infested cells. Based on these data, we observed a great variability of SMR across the different honey bee populations, with a mean proportion of non-reproducing mites reaching an overall score of 32.8%, and close to 16% of colonies exceeding a score of 50% (from observations based on minimum 10 single-infested cells).

In a next step, the variation of mite reproduction success in different honey bee genotypes was explored further by examining at least 35 single-infested cells. The results showed that some genotypes performed significantly better than others with three of them (one each of caucasica, ligustica, and mellifera origin) exhibiting comparatively high scores. However, the number of colonies investigated was quite small, and additional experiments are needed to confirm this observation. Considering that on average between 5% and 20% of mite foundresses remain infertile in European honey bees [6], and mite reproduction rates ranging from 0.78 to 0.9 have been reported from mite-susceptible control colonies in previous studies [27,48], the present results indicate that a considerable proportion of the honey bee populations in Europe may hold the potential to select for increased resistance (*sensu* [26]) to *V. destructor.* This is also supported by the fact that the SMR scores of colonies originating from preselected stock were consistently and significantly higher than the scores of unselected genotypes. The present scores observed in both the French surviving population (0.47 ± 0.12) and the VSH hybrid genotype (0.57 ± 0.11) were in the range of previous results from the French population (0.59 ± 0.02) and that reported from the mite-surviving population from Gotland (0.48 ± 0.02) [27,28].

### 4.2. Factors Affecting Measurement of SMR

While the present results indicate that honey bee colony resistance to *V. destructor* in many European bee populations could indeed be improved by selection for increased SMR, several factors may present a challenge towards the creation and implementation of such a selection approach. Obtaining a reliable estimate of the ability of a given colony to suppress mite reproduction is difficult and labor intensive. In addition, the score can be influenced by a number of different factors, such as the amount of worker and drone brood available [49] or the mite load of the colonies. The number of offspring per mite tends to decrease with high infestation levels [50], which may bias observed SMR scores towards increased values. Despite high infection rates found in some colonies (up to 80% in the brood), no correlation was observed between mite loads and SMR scores in the present study. However, such effects are complex and need to be further explored as they can possibly cancel each other out. For instance, colonies with high SMR expression may regulate the total amount of *V. destructor* in the colony, while colonies with low mite infestations may not trigger behaviors resulting in high SMR values. Further studies are necessary to confirm if SMR can also be influenced by other environmental factors, similarly to what has been shown for hygienic behavior and food availability, or virus infections [51,52,53]. In addition, although SMR has been described as a heritable trait [30,54], heritability estimates are currently unavailable for any population.

The reliability of the SMR score is highly dependent on the number of single-infested cells that are opened and assessed. While the best estimate for the score of a given colony requires the assessment of a high number of single-infested brood cells, as close as possible to the total number of such cells in the colony, this is obviously not a practicable approach. In previous studies, the number of observations per colony was not exactly specified but typically varied between 10 and 35 [27,49,55]. To evaluate the reliability of scoring we performed simulation analyses based on different numbers of observations and different levels of SMR. The results showed that estimation of the SMR score based on small numbers of single-infested cells varied widely, and scoring based on ten observations may lead to under- or overestimation of the true SMR score in the range of 30%. Even when the scoring was based on 35 single-infested cells, there remained considerable variation around the true SMR score.

Repeated measurements as is done for VSH [56] could increase reliability, however, the time window for SMR scoring is very narrow, which adds another level of complexity to applying this trait for selection. In most colonies, mite infestation levels in the brood can be very low early in the season [57], and a level that enables assessment of SMR with manageable input of time and labor is only possible after the peak of development, in the short period between late summer and early fall. To complicate matters further, as a high expression of the SMR trait results in a decrease of brood infestation, in such colonies it may become increasingly difficult to find enough single-infested cells for a reliable scoring even late in the season. One possible alternative could be to measure SMR in brood frames that were exposed to high infestation levels by placing them into mite-donor colonies during the open stage. However, this alternative procedure does not really reflect the natural situation, and the introduction of mites from a foreign origin may result in a biased score [34]. Together, these challenges may lay behind the reluctance of breeders to integrate and use the SMR trait in selection programs [58].

### 4.3. Triggers for SMR

The development of a simple bioassay to score the SMR potential of a colony is a challenging task, and the factors responsible for SMR require further investigation. Several different mechanisms may trigger the SMR phenotype and may originate from host and/or parasite features. Host factors seem to be central in some populations, as a change of queen can lead to a change in SMR phenotype [59]: (i) SMR can indirectly result from varroa-sensitive hygiene (VSH) behavior, when adult bees preferentially target brood infested by reproducing mites [34], but leave brood cells containing non-reproducing mites untouched. (ii) Female mites escaping from VSH-targeted cells may survive and enter a new cell for reproduction, but due to their previous aborted reproduction cycle, could face an increased risk of reproduction failure. This mechanism remains to be confirmed, even if a high correlation between the level of VSH and SMR was found in some populations [34,60,61]. (iii) Recapping, which consists of the opening and subsequent recapping of the targeted cells by the bees, may also influence the reproductive capacity of mites within the targeted brood. In an artificial uncapping/recapping experiment, it was shown that targeted cells have a lower varroa reproduction rate [35], and naturally surviving populations displaying increased SMR scores also display high recapping rates [21,35]. (iv) Physiological or behavioral features of the brood itself may also influence the ability of varroa to reproduce [36,37,38], even though the exact mechanisms through which the brood may impair varroa reproduction remain unknown.

Nonetheless, parasite features may also influence varroa reproduction. Recent research has shown that genetic variation in mites is higher than previously assumed [39,40], and such results may contribute to an improved understanding of the interactions between the genotypes of host colonies and their parasites. The physiological status of mites invading cells could also play a significant role. For instance, there are strong indications that mite reproductive success may be reduced after prolonged periods on adult bees without access to brood. Otten [62] describes significant seasonal differences in mite reproductive success, with lowest levels (70%) in late winter and high values (up to 90%) in July. Recent research also indicates that mite reproduction success decreases after broodless periods, for instance caused by prolonged caging of the queen or application of trapping combs as integrated varroa control measures [63], or in the context of swarming [64].

The failure of mites to reproduce, regardless if depending on host and/or parasite mechanisms or environmental factors, can be characterized by three different features of mite reproduction: infertility of the mite, i.e., total absence of offspring, absence of the male, which will prevent the mating of the female offspring, or a delay in egg laying and/or offspring development which will prevent mites from reaching the adult stage before the developing bee emerges. Each of these features may potentially be linked more specifically to one or more host/parasite mechanisms regulating varroa reproduction. In this study, the three possible features were identified in all investigated populations, with the delay being the most frequent reason for mite reproduction failure. In France, the proportion of male absence was higher than in all other populations studied, while infertility was particularly low in Moldova and Poland (Figure 6B). The proportions of the three features that form SMR do not vary according to the SMR level of the colony, suggesting that all three features may be important to support SMR. The history of host-parasite interactions may have shaped the different mechanisms observed in this study. Further studies would be necessary to understand the link between the mite reproduction features, and the host-parasite mechanism that regulate mite reproduction.

### 4.4. Comparing SMR to Other Means of Selection for Varroa Resistance

The considerable amount of work involved in scoring SMR, and as we have shown, a considerable level of variability in measurement, can question the advantages of this trait compared to other traits known to be related to resistance. The assays currently available to assess the detection and uncapping of varroa parasitized brood through VSH behavior equally are time-consuming and more restrictive than the SMR assay in terms of brood and mite requirements, and also display high levels of variability in the outcome [56]. The development of a bioassay allowing to study the bees’ behavior without the need to have mites to infest the cells would facilitate the study of both SMR and VSH traits. The development of molecular markers are a great hope in this direction, but despite several recent findings [24] no commercial service is currently available.

The advantage of the SMR phenotype is that it encompasses several possible mechanisms leading to resistance, such as action from the adult bees through VSH or impairment of varroa reproduction by the brood. An alternative and even more straightforward method of resistance evaluation would be to follow the growth of the varroa population in colonies over time. It can be done with far less work than VSH or SMR scoring, and colonies with a lower growth during the season are expected to survive better than those with more mites. The value of this approach is however hampered, since many environmental factors can influence the varroa load in a colony, such as the influx of mites from neighboring colonies [65], which probably explains the low heritability of varroa population growth [30,66].

Letting nature do the selection in order to breed from the surviving colonies, sometimes referred to as the Bond method [18] or Darwinian beekeeping [67], has gained attention and some attractiveness due to the repeated finding of populations able to survive without treatments [17,19,68]. As discussed above, however, so far attempts to bring such honey bees bred from “natural selection” into beekeeping on a wider scale have failed.

## 5. Conclusions

SMR stands as a complex trait, as it can be triggered by several host and/or parasite mechanisms, influenced by a wide variety of environmental factors, and remains challenging to phenotype accurately in the field. In the present study, it was not possible to identify the specific mechanism underlying the SMR trait, and we found large uncertainty in its estimation when few cells are investigated. Nevertheless, SMR implies lower mite population growth and, thus, remains a trait of great importance for the development of selection strategies to improve the ability of honey bee colonies to fight infestation by one of the most important honey bee enemies, the mite *V. destructor*.

## Figures and Tables

**Table 1:** Sampling strategy throughout Europe and corresponding genotypes.

Country	Laboratory	Genotypes	Samples (≥10 SIC)	Samples (≥35 SIC)
Croatia (Hr)	HPA	*carnica*	12	7
	OS	*carnica*	16	12
Denmark (Dk)	Dk	Buckfast	6	2
France (Fr)	INRA	Buckfast, *mellifera* mix, VSH	56	34
	ITSAP	Buckfast, *caucasica*, *carnica* mix, *ligustica* mix	39	33
Germany (De)	LLH Bee Institute	*carnica*	105	35
Greece (Gr)	HAO-API	*cecropia*, *macedonica*	15	0
Italy (It)	CREA-API	*ligustica*	12	2
	Aspromiele	Buckfast	20	0
Lithuania (Lit)	Vilnius	*carnica* mix	10	0
R. of Moldova (Mol)	IZASM	*carpatica*	23	13
Norway (Nor)	NBA	*mellifera*	10	1
Poland (Pol)	Pulawy	*carnica*, *caucasica*, *mellifera*	17	7
	Olsztyn	*carnica*	29	9
Portugal (Por)	CIMO	*iberiensis*	7	0
Romania (Rom)	ICDA	*carpatica*	23	19
Switzerland (Sw)	Liebefeld	*carnica* mix	14	2
**Total**			**414**	**176**

SIC: single-infested brood cells.

## Supplementary Materials

The following are available online at https://www.mdpi.com/2075-4450/11/9/595/s1, Table S1: Simulation results for the precision of the SMR estimates.
