# Supplementary material for: Evaluation of Suppressed Mite Reproduction (SMR) Reveals Potential for Varroa Resistance in European Honey Bees (Apis mellifera L.)
Source: Insects. 2020 Sep 3;11(9):595. doi: 10.3390/insects11090595 (PMC7565386; doi:10.3390/insects11090595)
Supplement: Supplementary file 1 [file insects-11-00595-s001.pdf]

Supplementary Material

Table 1. Simulation results for the precision of the SMR estimates.

I	S	Loq	Hiq	Mae	Rmse	Med	M
**10**	0.20	0.01	0.58	0.129	0.166	0.22	0.25
**10**	0.35	0.06	0.74	0.148	0.183	0.36	0.37
**10**	0.50	0.14	0.85	0.154	0.188	0.49	0.50
**10**	0.70	0.29	0.95	0.144	0.178	0.68	0.67
**35**	0.20	0.06	0.41	0.074	0.093	0.20	0.22
**35**	0.35	0.15	0.57	0.087	0.109	0.35	0.36
**35**	0.50	0.28	0.71	0.091	0.113	0.50	0.50
**35**	0.70	0.47	0.87	0.084	0.105	0.69	0.69
**100**	0.20	0.10	0.32	0.044	0.056	0.20	0.21
**100**	0.35	0.22	0.48	0.053	0.066	0.35	0.35
**100**	0.50	0.36	0.63	0.055	0.069	0.49	0.50
**100**	0.70	0.56	0.81	0.051	0.064	0.69	0.70

Statistics of the posterior distribution of the SMR estimates are given for varying values of the number of single-infested cells (i) and true SMR value (s); The statistics are: loq, 2.5% quantile; hiq, 97.5% quantile, mae: mean average error, rmse: root mean squared error, med: median, m: mean.
